# Supplementary material for: Clinal genomic analysis reveals strong reproductive isolation across a steep habitat transition in stickleback fish
Source: Nat Commun. 2021 Aug 11;12:4850. doi: 10.1038/s41467-021-25039-y (PMC8358029; doi:10.1038/s41467-021-25039-y)
Supplement: Supplementary file 1 — Supplementary Information [file 41467_2021_25039_MOESM1_ESM.pdf]

Supplementary Information for:

Clinal genomic analysis reveals strong reproductive isolation across a steep habitat transition in stickleback fish

Quiterie Haenel[1]*, Krista B. Oke[2,3], Telma G. Laurentino[1], Andrew P. Hendry[3] and Daniel Berner[1]*

[1] Department of Environmental Sciences, Zoology, University of Basel, Basel, Switzerland

[2] College of Fisheries and Ocean Sciences, University of Alaska Fairbanks, Juneau, Alaska

[3] Redpath Museum and Department of Biology, McGill University, Montreal, Quebec, Canada

*corresponding authors: quiterie.haenel@unibas.ch, daniel.berner@unibas.ch

Contents

Supplementary Figures

Supplementary Fig. 1 Geometric morphometric analysis of lake, marsh and stream stickleback. (Pages 4-5)

Supplementary Fig. 2 Genetic differentiation between Misty Lake and inlet stream stickleback across all chromosomes. (Pages 6-8)

Supplementary Fig. 3 Distribution of differentiation between the sites L1 and S7 across all genome-wide SNPs. (Page 9)

Supplementary Fig. 4 Genomic differentiation along the lake-stream transition at the loDiff SNPs. (Page 10)

Supplementary Fig. 5 Raw distribution of cline center location and cline width estimates. (Page 11)

Supplementary Fig. 6 Simulation study to assess the influence of the magnitude of differentiation between two contiguous populations on cline parameter estimation. (Pages 12-13)

Supplementary Fig. 7 Genetic cline modeling with the two terminal sites of the geographic gradient excluded. (Pages 14-15)

Supplementary Fig. 8 Pairwise differentiation along a chromosome, expressed by F_{ST}. (Page 16)

Supplementary Fig. 9 Robustness checks of the simulations of divergence with gene flow across a habitat transition. (Page 17)

Supplementary Fig. 10 Characterization of the marsh habitat in the Misty system. (Page 18)

Supplementary Fig. 11 Exploring the approximate proportion of migrants from the lake into the marsh during the flood. (Page 19-20)

Supplementary Fig. 12 Genetic differentiation between the study sites. (Page 21)

Supplementary Fig. 13 Alternative analysis of chromosome center-biased differentiation (CCBD). (Page 22)

Supplementary Fig. 14 Determining an appropriate number of generations for the individual-based simulations. (Page 23)

Supplementary Tables

Supplementary Table 1 Characterization of the study sites in the Misty Lake watershed. (Page 24)

Supplementary References (Page 25)

Supplementary Figures

Supplementary Fig. 1

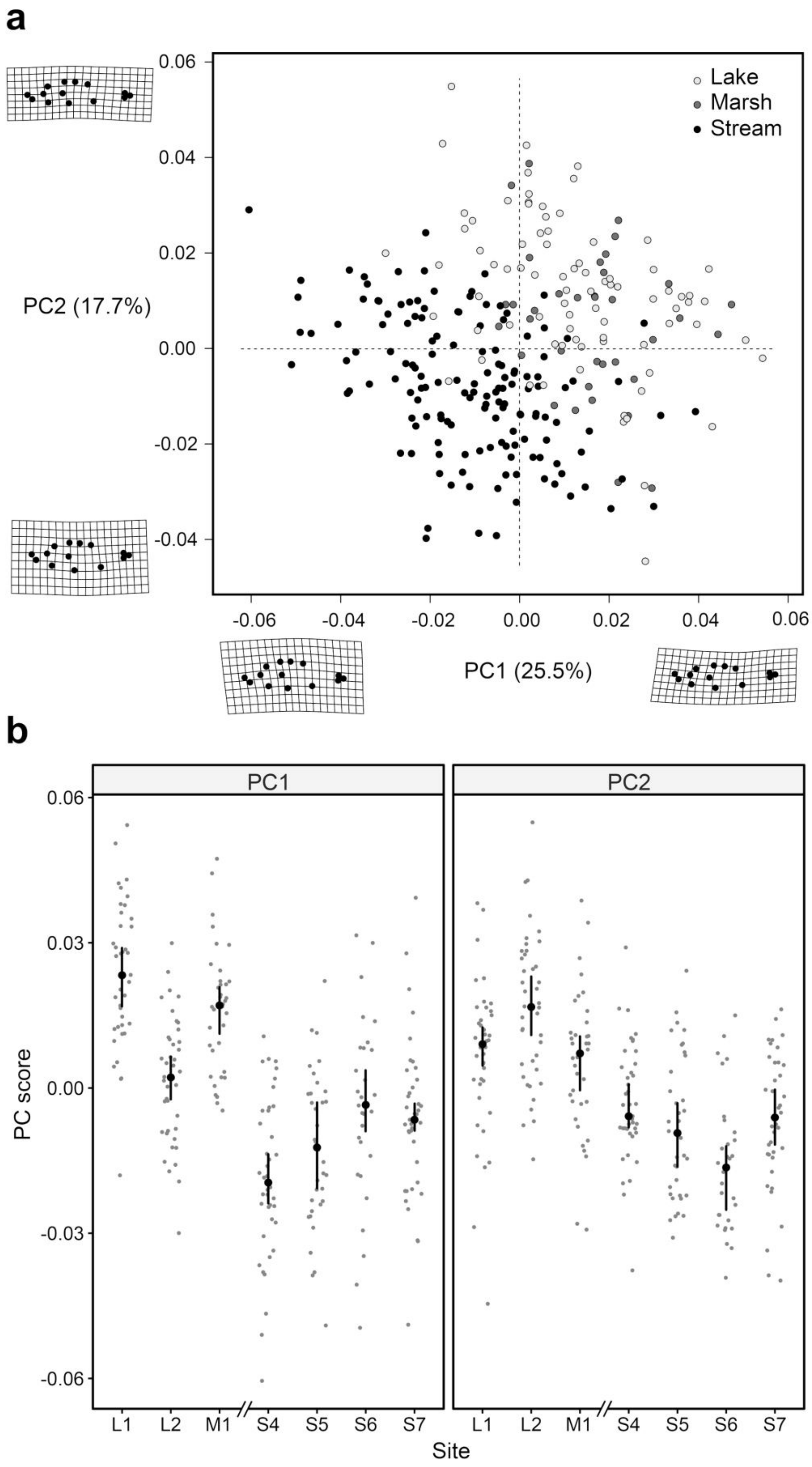

Supplementary Fig. 1 Geometric morphometric analysis of lake, marsh and stream stickleback. a Each point represents an individual fish along the first two principal components (PC1, PC2) obtained by landmark-based shape analysis (methodological details given in refs. [1,2]). Individuals are color coded according to their habitat (lake, marsh, inlet stream). The deformation grids visualize the body shape associated with the lowest and highest observed score along each PC. **b** Individual PC scores shown separately for each study site (n = 40 individuals per site). Black dots and vertical lines represent site medians with their bootstrap 95% compatibility interval. Note that both PCs capture variation in body depth, that stream fish tend to exhibit deeper bodies than lake fish, and that marsh fish resemble lake fish.

Supplementary Fig. 2

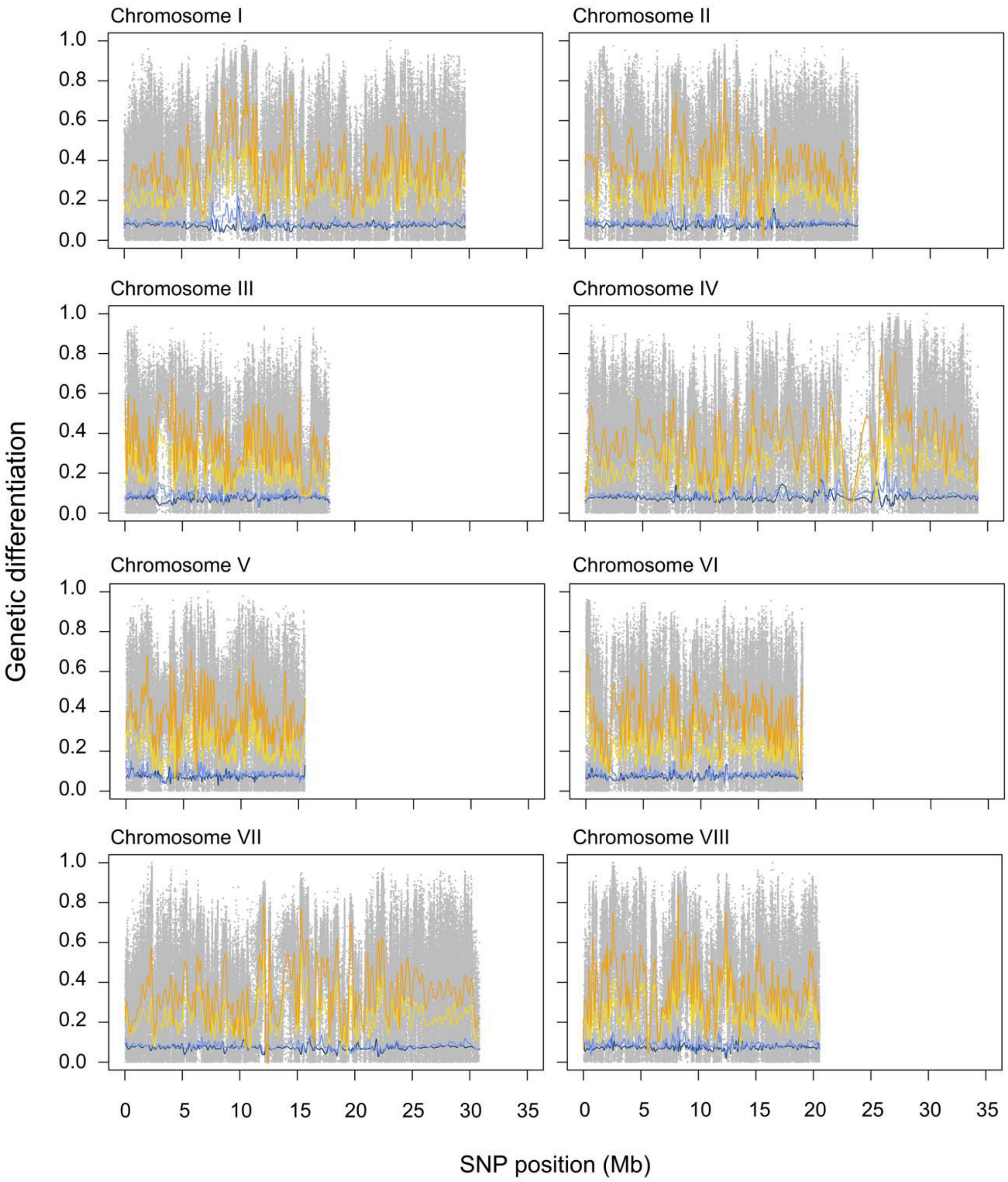

Supplementary Fig. 2

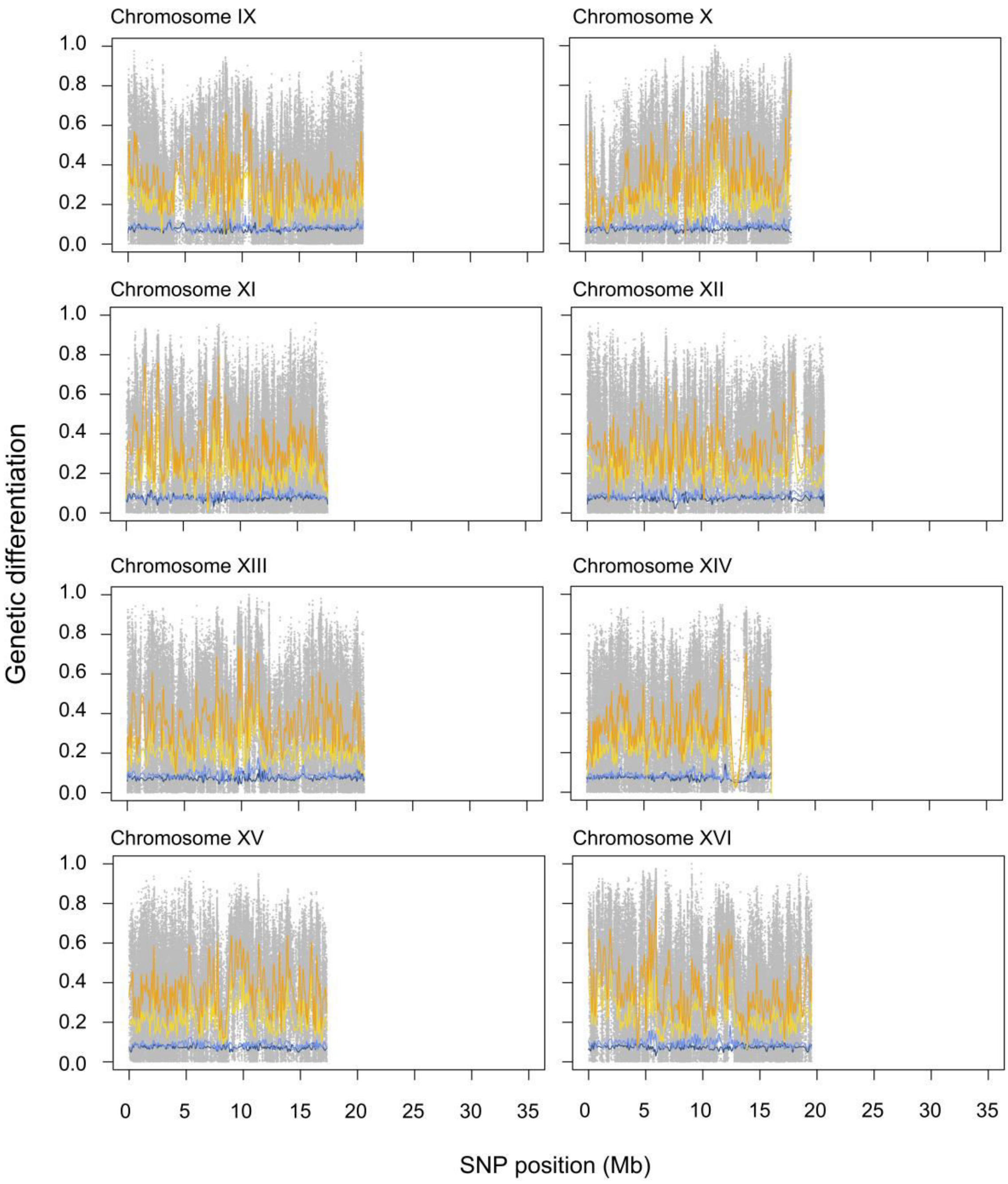

Supplementary Fig. 2

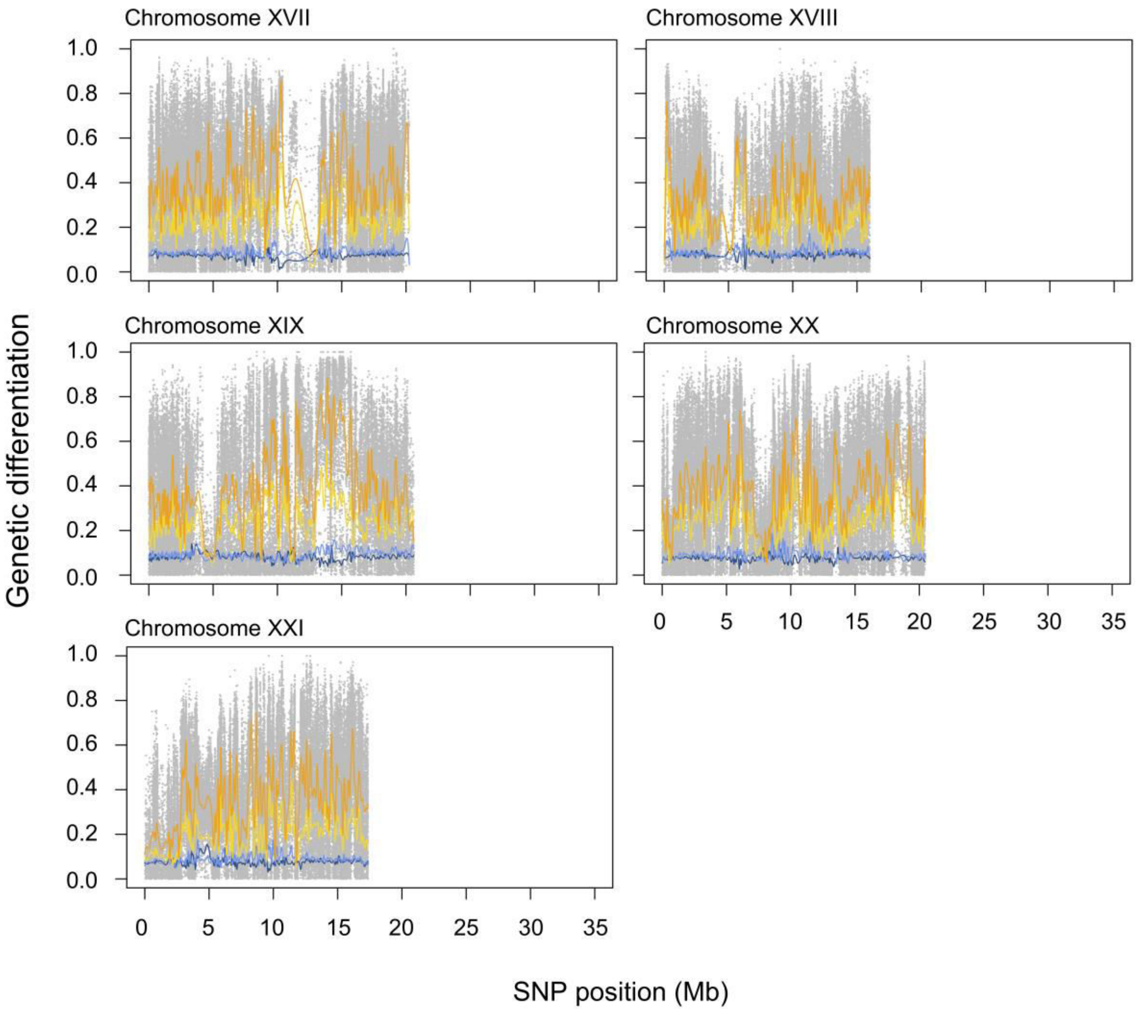

Supplementary Fig. 2 Genetic differentiation between Misty Lake and inlet stream stickleback across all chromosomes. Differentiation is expressed by the absolute allele frequency difference AFD. The presentation format follows that of Fig. 3.

Supplementary Fig. 3

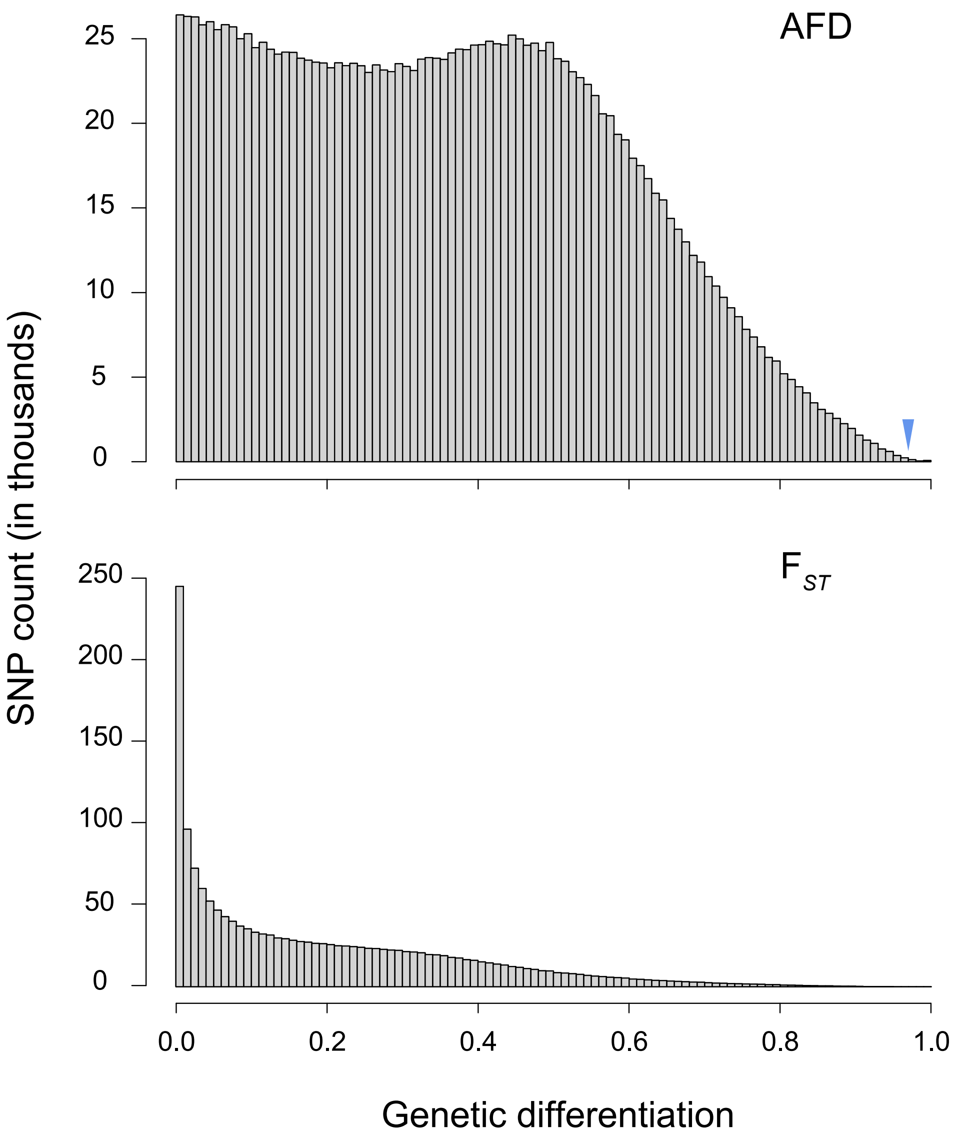

Supplementary Fig. 3 Distribution of differentiation between the sites L1 and S7 across all genome-wide SNPs. In the top panel, differentiation at 1,708,118 SNPs (including the sex chromosome) passing quality filtering thresholds for this specific sample comparison is expressed by the absolute allele frequency difference AFD. To facilitate comparison with previous work, the lower panel shows the analogous distribution based on an F_{ST} estimator (G_{ST}[3]). The genome-wide median differentiation is 0.352 (AFD) and 0.139 (F_{ST}). The blue triangle in the upper panel indicates the threshold AFD value of 0.97 that was applied to identify the panel of selected SNPs.

Supplementary Fig. 4

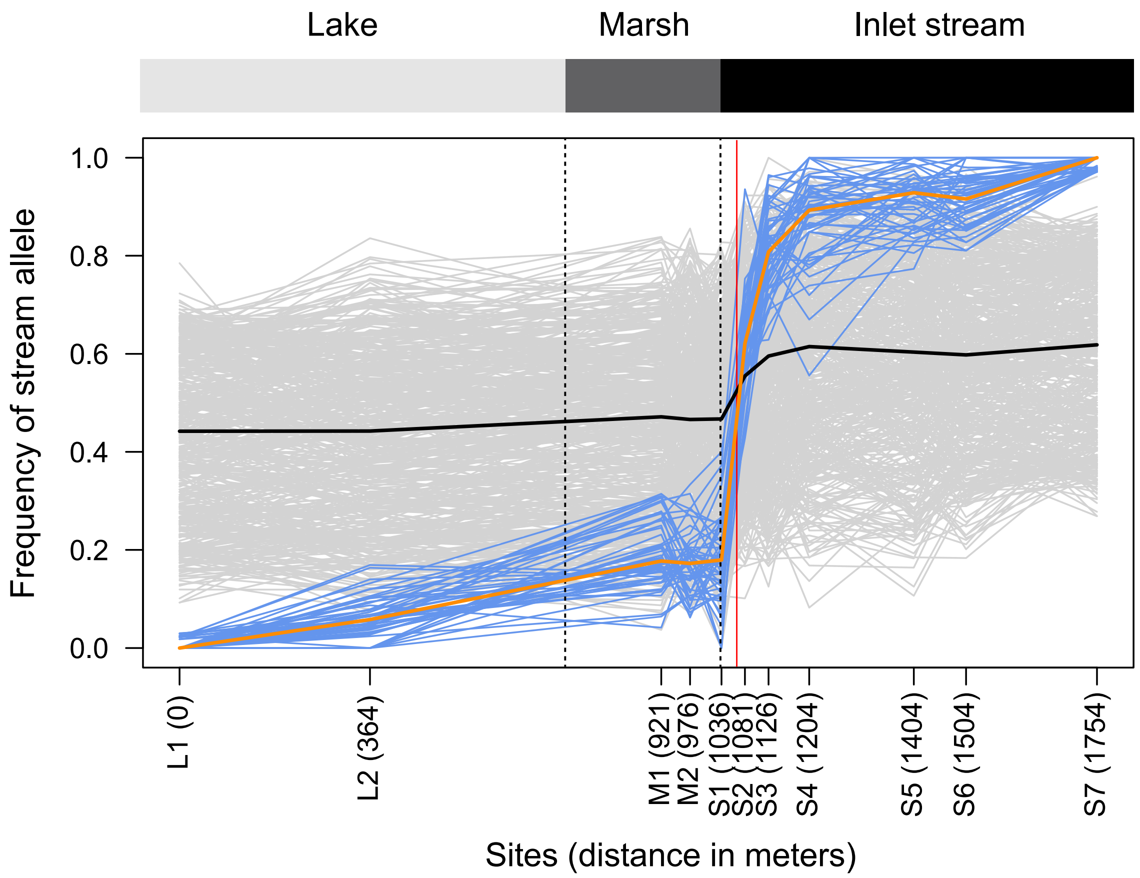

Supplementary Fig. 4 Genomic differentiation along the lake-stream transition at the loDiff SNPs. This graphic is analogous to Fig. 2b, except that the allele frequencies from the neutral SNP category are here replaced by the corresponding data from loDiff SNPs (gray and black lines). The loDiff SNPs are 500 markers chosen at random among all SNPs deviating by no more than 0.1% from half the genome-wide median AFD (c. 0.17) in the L1-S7 sample comparison. These SNPs are even less likely than the neutral SNPs to be influenced by divergent selection on physically close genome regions, hence capture genome-wide differentiation by drift between Misty Lake and inlet stream stickleback.

Supplementary Fig. 5

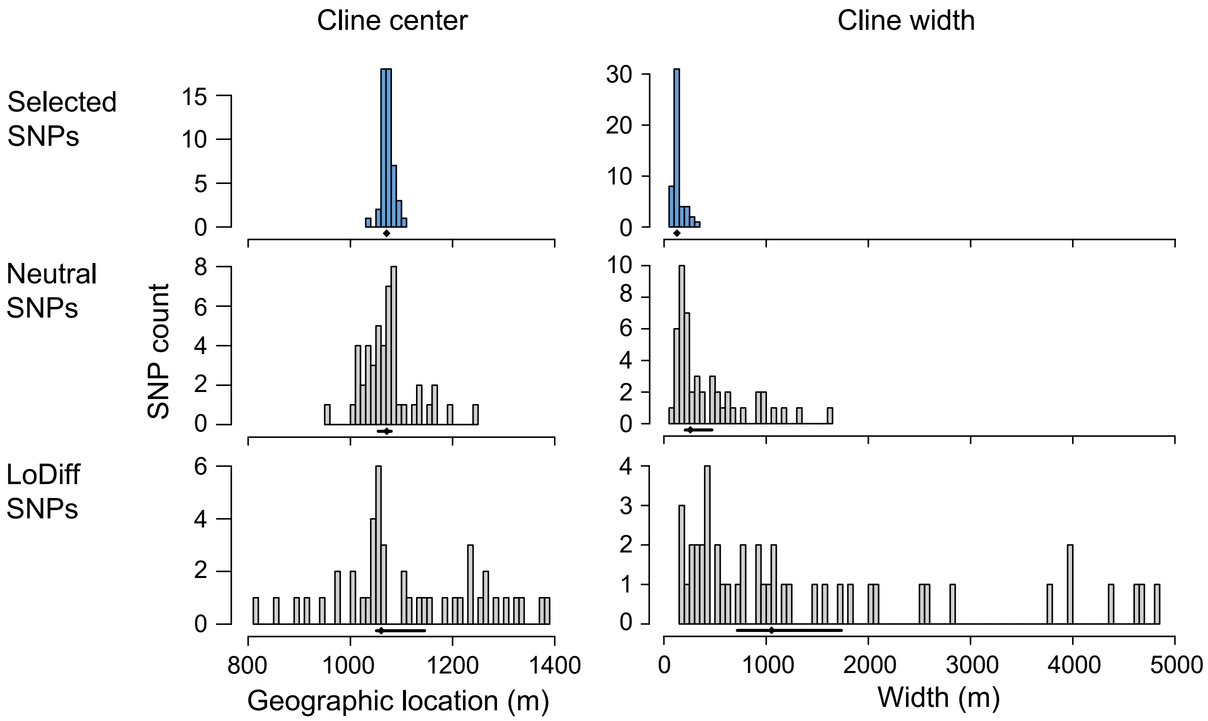

Supplementary Fig. 5 Raw distribution of cline center location and cline width estimates. The histograms show cline center and width estimates obtained by genetic cline modeling for each of the 50 individual SNPs from the three marker categories (selected SNPs in blue). The underlying data points are the medians across ten replicate model fitting runs for each SNP. Below each histogram, the median and the associated 95% bootstrap compatibility interval across the SNPs are visualized (values identical to those in Fig. 4). A single SNP from the neutral SNP panel displayed extremely spatially unstable allele frequencies compared to all other SNPs and was therefore considered a technical outlier and excluded from the histogram (center estimate 1301 m, width estimate 2701 m), although this SNP was included for the summary statistics (having a trivial influence). Also, to maintain a reasonably high visual resolution along the X-axis, five loDiff SNPs exhibiting extreme values for cline center (-458 m, 368 m, 431 m, 1516 m, 1655 m) and cline width (5229 m, 5321 m, 5471 m, 7919 m, 20766 m) were omitted from the histograms, although these SNPs were not considered technical outliers and were included for the calculation of summary statistics.

Supplementary Fig. 6

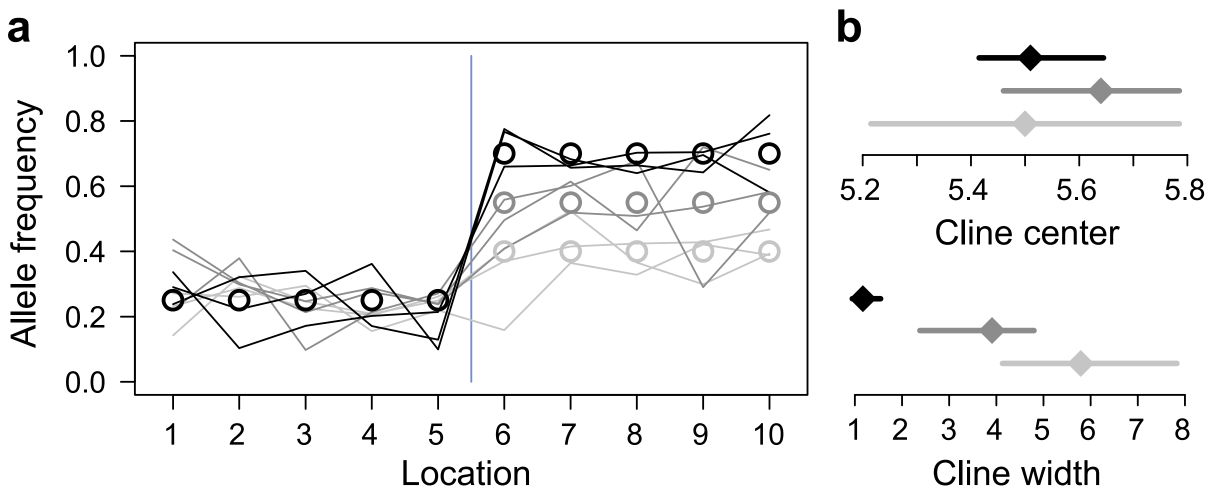

Supplementary Fig. 6 Simulation study to assess the influence of the magnitude of differentiation between two contiguous populations on cline parameter estimation. a We modeled two populations, each represented by five evenly spaced geographic locations. The boundary between the populations thus occurred between the locations 5 and 6 (vertical blue line) and coincided with a shift in expected allele frequencies. The magnitude of this shift (AFD) differed among simulations from 0.15 (light gray) to 0.30 (gray) and 0.45 (black; stronger differentiation would have produced allele frequencies exceeding the 0-1 range and was therefore avoided). The population on the left of the allele frequency breakpoint always had an expected frequency of 0.25, while the expected frequency of the population on the right of the breakpoint was obtained by adding the corresponding AFD (the expected frequencies at each location are shown as circles; on the left of the breakpoint, these are perfectly overlapping). To introduce stochasticity, we added a random draw from the normal distribution with a mean of zero and a standard deviation of 0.08 to the expected allele frequency at each site (this standard deviation produced variation in allele frequencies among locations qualitatively similar to the variation observed empirically). The expected magnitude of random noise and the true cline position and width were thus tightly controlled and invariant among the different simulation types, which differed only in AFD between the populations. The final allele frequency data obtained in this way (three randomly chosen examples are illustrated by lines for each AFD category) were then subject to cline parameter

estimation with HZAR[4], analogously to our empirical analysis. We here used the locations as distance values and consistently assumed a sample size of 100 underlying allele frequency estimation at any given location. The estimates for cline center and width from a single MCMC run per simulation replicate were then saved, with 30 such replicates run per AFD category. Data from the replicate simulations were used to compute median cline center and width and their 95% bootstrap compatibility intervals (CIs) based on 10,000 resamples. **b** This simulation experiment produced insights relevant to the interpretation of our empirical analysis: first, we found that across all three modeled levels of AFD between the two populations, cline center location was estimated accurately (top panel); the median estimate approximated the expected value of 5.5 closely, or the latter was at least well within the CI. However, estimation precision for cline center decreased (wider CIs) with decreasing AFD between the populations. The second observation was that median cline width and its spread increased with decreasing AFD (bottom panel). All these observations mirror patterns also emerging from our empirical analysis (Fig. 4). This leads us to conclude that the greater cline widths observed empirically for the neutral and especially the loDiff SNPs relative to the selected SNPs offer no evidence of geographically more extensive gene flow in the latter SNP categories. Even if reproductive isolation is complete, genome regions exhibiting greater population differentiation by chance (stronger drift) or due to divergent selection will produce lower cline width estimates. We note that this influence of the magnitude of AFD on cline parameters was observed even when fitting models to the precise population-specific allele frequencies from all locations (i.e., no random noise), when running models without tails, and when increasing the number of geographic locations on either side of the population boundary to ten (details not presented).

Supplementary Fig. 7

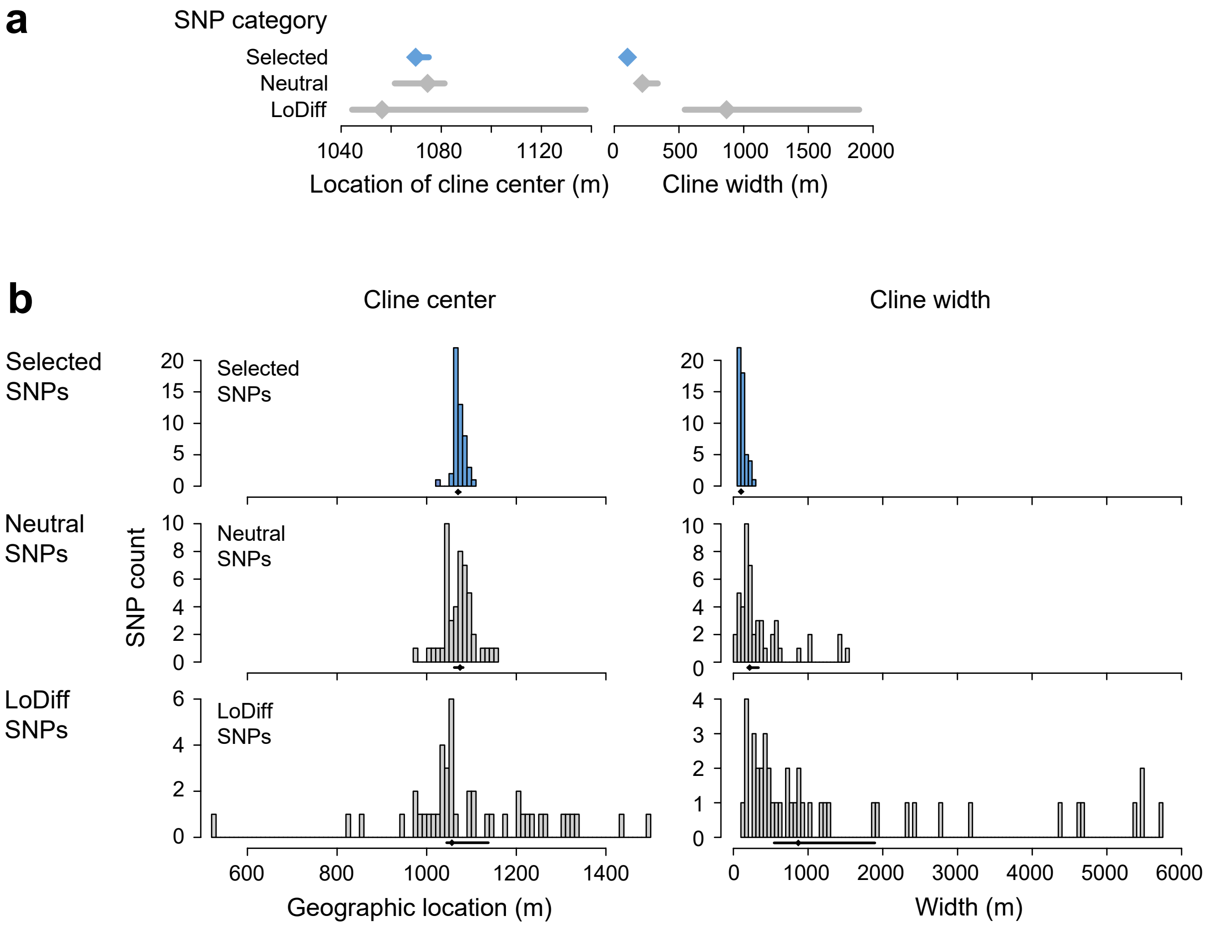

Supplementary Fig. 7 Genetic cline modeling with the two terminal sites of the geographic gradient excluded. To examine potential SNP ascertainment bias in cline model fitting, all modeling was here repeated with allele frequency data from the sampling sites L1 and S7 excluded, resulting in nine total sampling sites. The rationale was that AFD observed in the comparison of these two sites was used as the basis for delimiting the SNPs for the selected, neutral, and loDiff marker panels. The allele frequencies at L1 and S7 were thus not fully independent, contrary to all other sampling sites. Cline modeling with these reduced data sets was otherwise carried out as described for the analysis with the full 11 sites, and the same graphing conventions were followed (see Fig. 4 and Supplementary Fig. 5). Both the summary statistics **a** and the underlying distributions **b** of cline center and width estimates across SNPs for the three SNP categories are very similar to the corresponding

results with the sites L1 and S7 included (Fig. 4 and Supplementary Fig. 5) and support the same conclusions. In the histograms for the neutral SNPs, a single technical outlier marker was again excluded (estimated cline center 7099 m; cline width 140705 m), and for ease of presentation, a few loDiff SNPs showing extreme estimates for cline center (-2928 m, 254 m, 1711 m, 1946 m, 6086 m) and width (6226 m, 6341 m, 6559 m, 19400 m, 29821 m, 57318 m) are not visualized. However, all these SNPs were included for the calculation of the summary statistics in **a**.

Supplementary Fig. 8

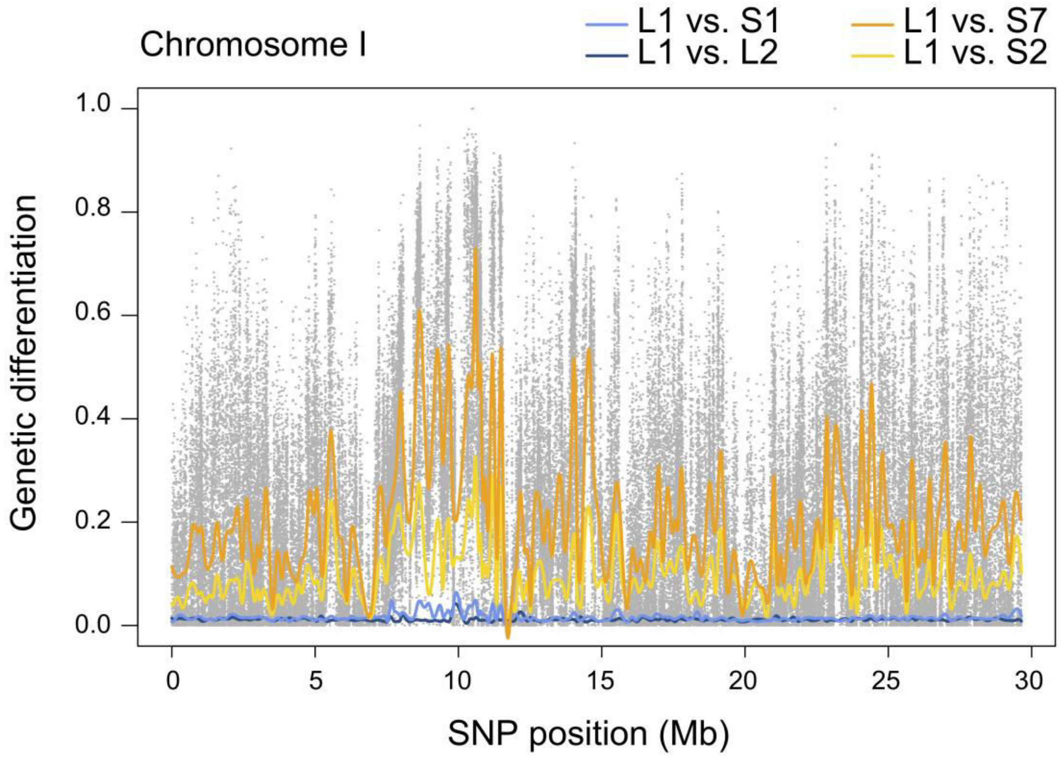

Supplementary Fig. 8 Pairwise differentiation along a chromosome, expressed by F_{ST}. The figure follows the same conventions as Fig. 3, except that differentiation at each SNP in each sampling site comparison was quantified by F_{ST} (the G_{ST} estimator of ref. [3]). Note that due to the low sensitivity of F_{ST} compared to AFD when population differentiation is weak or modest[5], the relatively strong differentiation peaks near 10 Mb in the L1-S1 site comparison are less obvious when differentiation is based on F_{ST} as opposed to AFD (compare to Fig. 3).

Supplementary Fig. 9

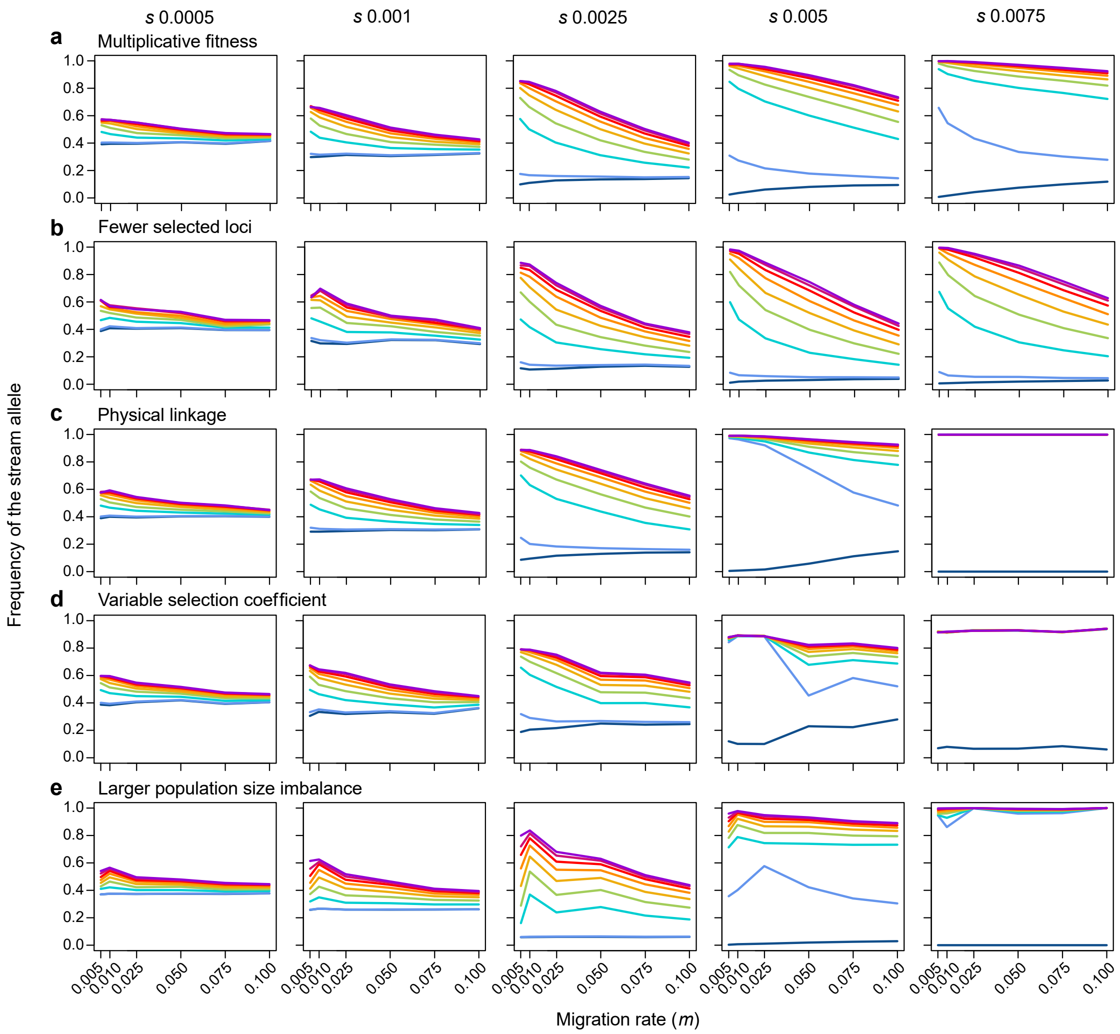

Supplementary Fig. 9 Robustness checks of the simulations of divergence with gene flow across a habitat transition. The presentation format follows Fig. 6, but the simulations were performed with the standard model modified in the following ways: **a** The loci contribute to fitness multiplicatively, as opposed to additively. **b** Only ten loci are under selection, as opposed to 100. **c** All loci are physically linked on a single chromosome exhibiting crossover, as opposed to free segregation. **d** The selection coefficients are not identical among loci, but are drawn from an exponential distribution (rate = 1/*s*). Note that the latter causes greater stochasticity in the simulation outcome, as particularly evident with s = 0.005. **e** The lake population size is ten times the stream population size.

Supplementary Fig. 10

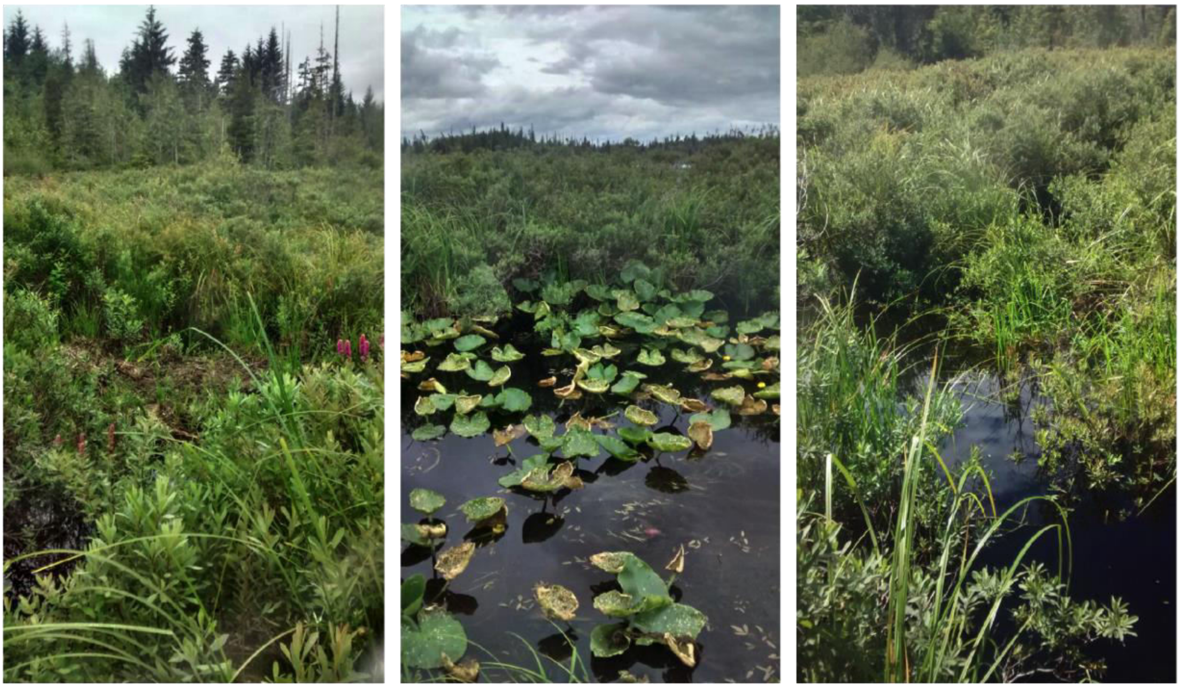

Supplementary Fig. 10 Characterization of the marsh habitat in the Misty system. The marsh represents the transition from the inlet stream (flowing through woodland) to the lake. This habitat is dominated by short (< 1.5 m), dense vegetation intersected by relatively narrow, deep channels. During most of the summer, one can walk on mats of this vegetation, the roots of which reach approximately 30 cm below the water surface. During the flood, these mats became entirely submerged, likely allowing fish to swim through vegetation that days before would have been above water. Supporting this view, stickleback catch rates at the marsh site increased dramatically during the flood (Krista B. Oke, unpublished data) (Photo credits: Krista B. Oke).

Supplementary Fig. 11

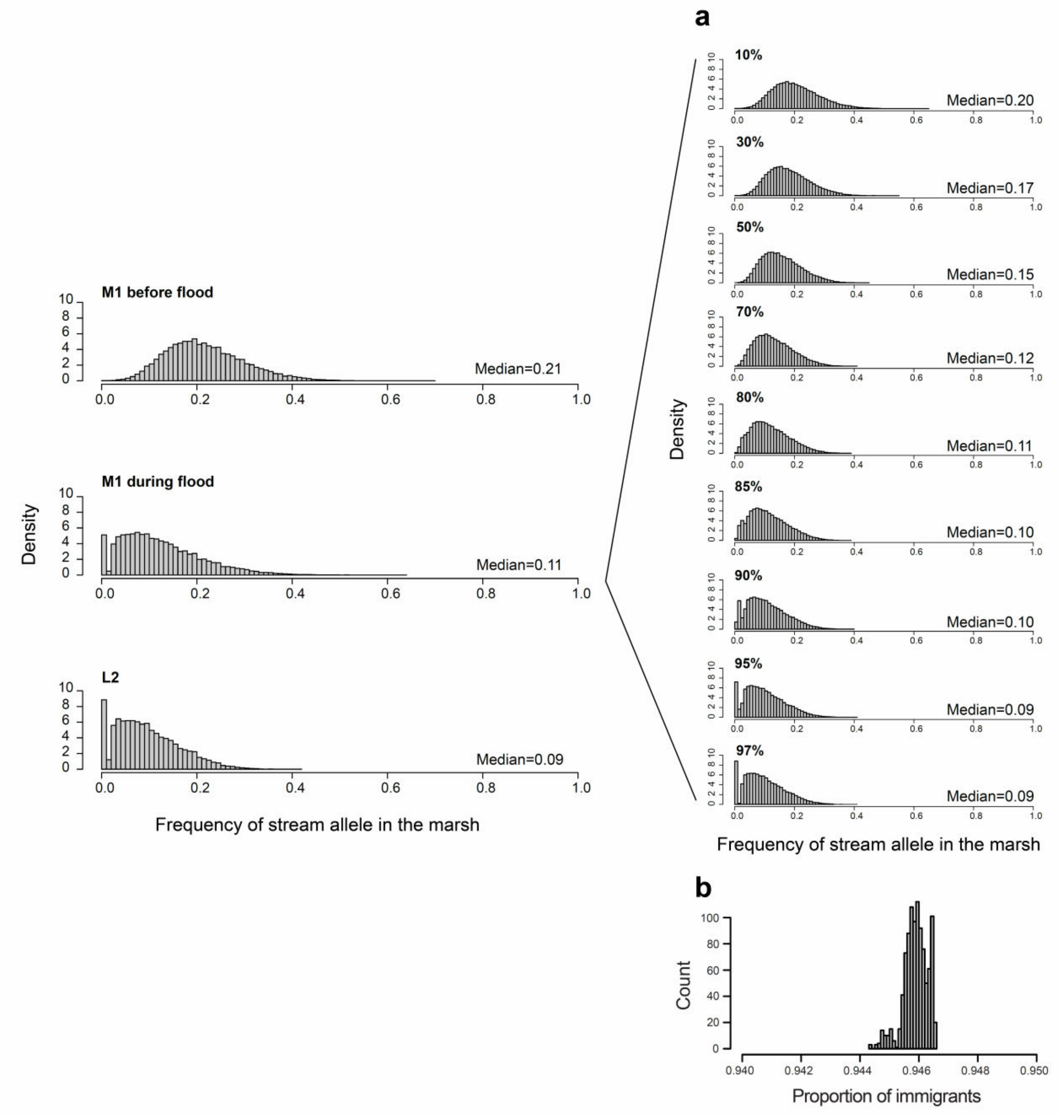

Supplementary Fig. 11 Exploring the approximate proportion of migrants from the lake into the marsh during the flood. This analysis focused on the same 49,677 SNPs highly differentiated between the lake and stream population also underlying Fig. 7. We here performed weighted averaging of the frequency of the stream allele at each SNP between the marsh sample (M1) before the flood (top left histogram) and the lake sample closest to this marsh site (L2; bottom left). The relative weight of each sample was varied to mimic different levels of dispersal of

lake fish into the marsh during the flood. We then asked what relative proportion of immigrant lake fish at the marsh site is required to yield a stream allele frequency distribution qualitatively resembling the distribution observed empirically during the flood (middle left). The evaluation of the resulting distributions was first performed visually, paying particular attention to the proportion of SNPs exhibiting a stream allele frequency very near zero. The allele frequency distributions resulting from nine exemplary immigrant proportions (indicated on top of each panel) are shown in **a**. This exploration suggested that the proportion of lake immigrants present at the marsh site during the flood was very high, likely around 90 - 95%. In addition, this proportion was estimated by approximate Bayesian computation (ABC). We here characterized the stream allele frequency distribution by using its 0.1, 0.5 and 0.9 quantiles as summary statistics. These statistics were calculated for 10,000 iterations, each using a relative proportion of immigrant lake fish drawn at random from the uniform distribution bounded between 0 and 1. The estimation of the proportion parameter was then performed with the *abc* R package[6], using a tolerance of 0.1 and the neural networks-based method for constructing the posterior distribution. The weighted median of the posterior distribution shown in **b** was 94.6% (2.5 and 97.5 percentiles: 0.9455, 0.9465), in good agreement with our visual estimation. Qualitatively similar results were obtained when using the simple rejection algorithm for generating the posterior distribution, and across the full range of tolerance values explored (0.2 - 0.01); the median of the posterior distribution was always between 0.80 and 0.95.

Supplementary Fig. 12

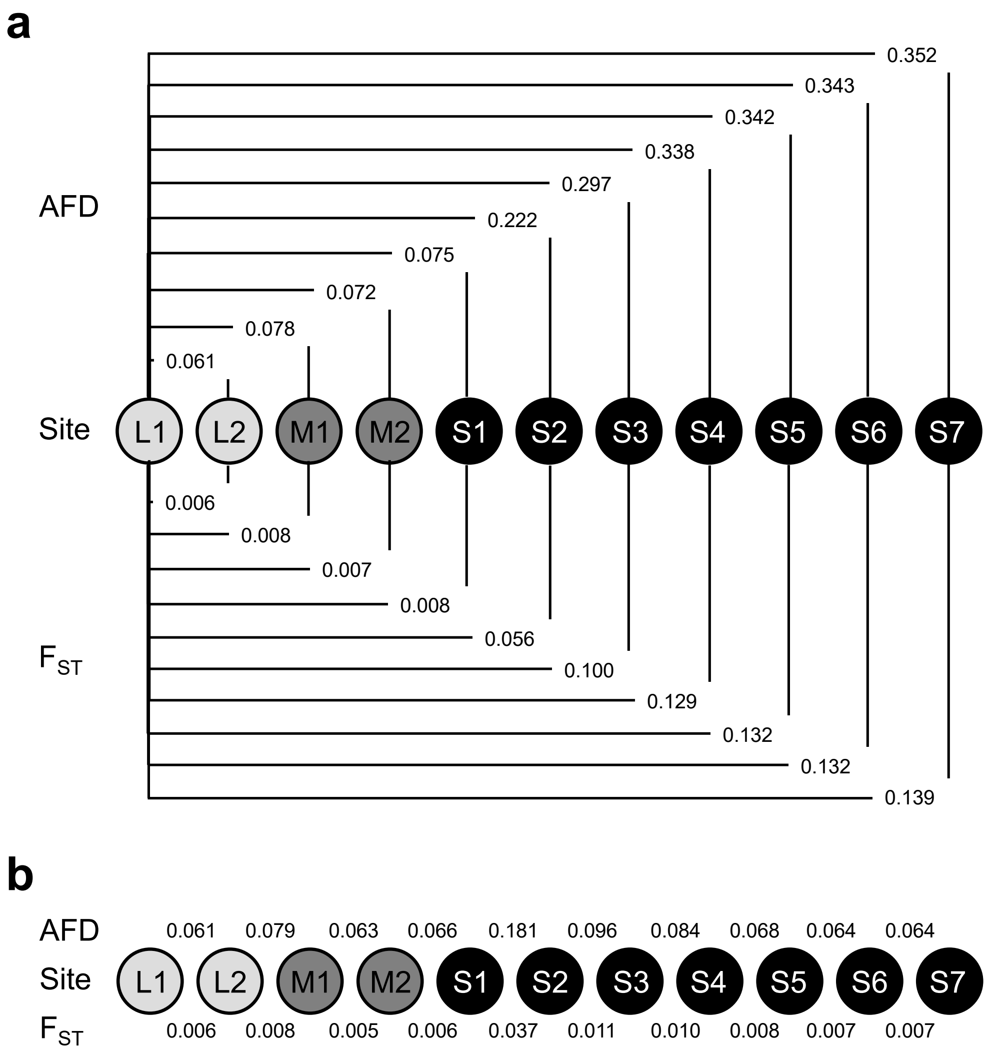

Supplementary Fig. 12 Genetic differentiation between the study sites. Median genetic differentiation, expressed by the absolute allele frequency difference AFD and F_{ST} (the estimator G_{ST} of ref. [3]), across all genome-wide SNPs (including the sex chromosome). Differentiation was calculated for all pairwise comparisons between L1 and each of the other sample sites **a**, and for all pairwise combinations of neighboring sample sites along the geographic gradient **b**.

Supplementary Fig. 13

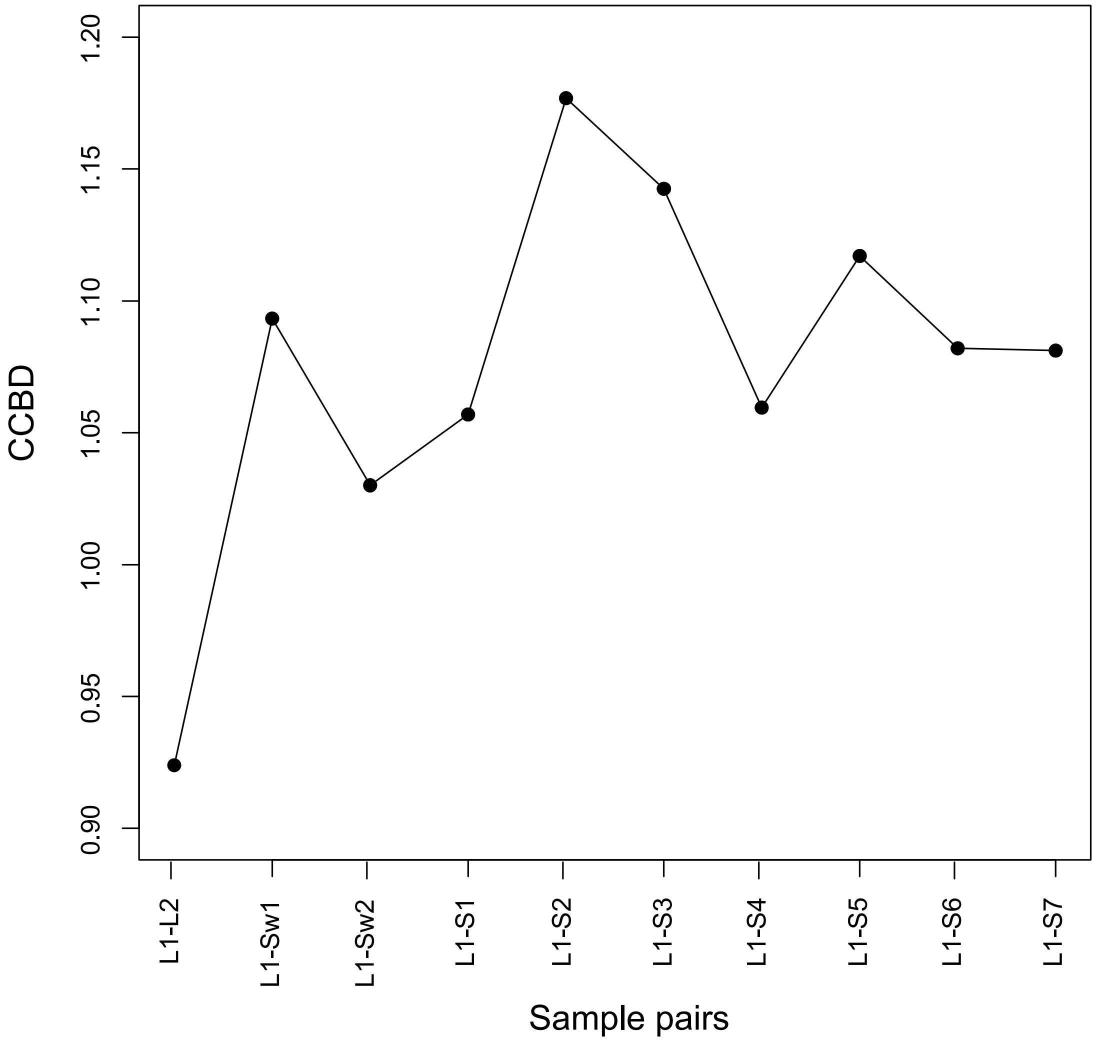

Supplementary Fig. 13 Alternative analysis of chromosome center-biased differentiation (CCBD). CCBD is here calculated as described in the Methods, but the samples used for pairwise comparison were not from adjacent sites as in Fig. 2c, but involved all combinations of the samples L2 to S7 with the sample L1. Consistent with Fig. 2c, the magnitude of CCBD is greatest for the L1-S2 sample pair, indicating selection-gene flow antagonism in the lowest reach of the inlet stream.

Supplementary Fig. 14

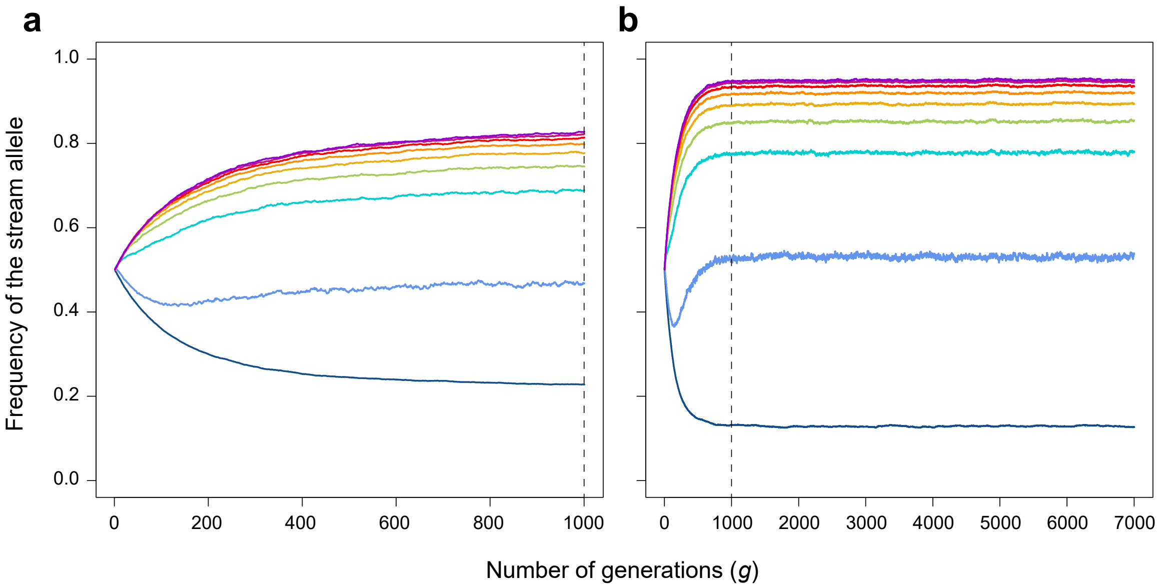

Supplementary Fig. 14 Determining an appropriate number of generations for the individual-based simulations. Simulations were run with our standard stepping stone model over **a** 1000 and **b** 7000 generations for an exemplary migration rate and selection coefficient combination (*m* 0.05 and *s* 0.005). Shown is the frequency of the stream allele over time averaged over 20 simulation replications. The dotted line indicates generation 1000 in both graphs. This exploration indicates that running our simulation model over 1000 generations allows the system to approach migration-selection balance.

Supplementary Tables

Supplementary Table 1 Characterization of the study sites in the Misty Lake watershed. Habitat type, GPS coordinates (in decimal degrees), and the number of individuals for the genomic and morphometric analyses are given for each site. For the genomic sample sizes, the values in parentheses indicate median read depth across all genome-wide positions. The site M1 was sampled at three different time points.

Site	Habitat	Latitude	Longitude	N genomics	N morphometrics
L1	Lake	50.60507824	-127.2685989	62 (103)	40
L2	Lake	50.604347	-127.262569	56 (80)	42
M1	Marsh	50.60516595	-127.2579478	50 (133)	36
M1 During the flood				56 (79)	-
M1 One year later				56 (114)	-
M2	Marsh	50.605087	-127.257812	56 (106)	-
S1	Stream	50.604618	-127.257198	56 (74)	-
S2	Stream	50.604414	-127.256683	56 (93)	-
S3	Stream	50.604375	-127.256141	56 (51)	-
S4	Stream	50.603808	-127.255397	40 (103)	41
S5	Stream	50.603056	-127.252444	52 (120)	37
S6	Stream	50.60223555	-127.2507798	56 (112)	33
S7	Stream	50.60060871	-127.2476535	50 (107)	41

Supplementary References

1. Kaeuffer, R., Peichel, C. L., Bolnick, D. I. & Hendry, A. P. Parallel and nonparallel aspects of ecological, phenotypic, and genetic divergence across replicate population pairs of lake and stream stickleback. *Evolution* **66**, 402-418 (2012).

2. Oke, K. B., Bukhari, M., Kaeuffer, R., Rolshausen, G., Rasanen, K., Bolnick, D. I. & Hendry, A. P. Does plasticity enhance or dampen phenotypic parallelism? A test with three lake-stream stickleback pairs. *J. Evol. Biol.* **29**, 126-143 (2016).

3. Nei, M. Analysis of gene diversity in subdivided populations. *PNAS USA* **70**, 3321-3323 (1973).

4. Derryberry, E. P., Derryberry, G. E., Maley, J. M. & Brumfield, R. T. HZAR: hybrid zone analysis using an R software package. *Mol. Ecol. Res.* **14**, 652-63 (2014).

5. Berner, D. Allele frequency difference *AFD* – an intuitive alternative to F_{ST} for quantifying genetic population differentiation. *Genes* **10**, 308 (2019).

6. Csilléry, K., François, O. & Blum, M. G. B. abc: an R package for approximate Bayesian computation (ABC). *Methods Ecol. Evol.* **3**, 475–79 (2012).
