# Supplementary material for: Clinal genomic analysis reveals strong reproductive isolation across a steep habitat transition in stickleback fish
Source: Nat Commun. 2021 Aug 11;12:4850. doi: 10.1038/s41467-021-25039-y (PMC8358029; doi:10.1038/s41467-021-25039-y)
Supplement: Supplementary file 3 — Description of Additional Supplementary Files [file 41467_2021_25039_MOESM3_ESM.docx]

Description of Additional Supplementary Files

Title: Supplementary Data 1

Description: Excel file listing the candidate genes around the 50 selected SNPs.

Title: Supplementary Software

Description: The command line settings for short read alignment, and all custom code written for data analysis in the R language are provided as Supplementary Software.
