# Supplementary material for: Clinal genomic analysis reveals strong reproductive isolation across a steep habitat transition in stickleback fish
Source: Nat Commun. 2021 Aug 11;12:4850. doi: 10.1038/s41467-021-25039-y (PMC8358029; doi:10.1038/s41467-021-25039-y)
Supplement: Supplementary file 6 — Reporting Summary [file 41467_2021_25039_MOESM6_ESM.pdf]

nature research

Corresponding author(s): Quiterie Haenel
Daniel Berner

Last updated by author(s): Jul 11, 2021

Reporting Summary

Nature Research wishes to improve the reproducibility of the work that we publish. This form provides structure for consistency and transparency in reporting. For further information on Nature Research policies, see Authors & Referees and the Editorial Policy Checklist .

Statistics

For all statistical analyses, confirm that the following items are present in the figure legend, table legend, main text, or Methods section.

n/a	Confirmed	
☐	☒	The exact sample size (*n*) for each experimental group/condition, given as a discrete number and unit of measurement
☐	☒	A statement on whether measurements were taken from distinct samples or whether the same sample was measured repeatedly
☒	☐	The statistical test(s) used AND whether they are one- or two-sided<br>*Only common tests should be described solely by name; describe more complex techniques in the Methods section.*
☒	☐	A description of all covariates tested
☐	☒	A description of any assumptions or corrections, such as tests of normality and adjustment for multiple comparisons
☐	☒	A full description of the statistical parameters including central tendency (e.g. means) or other basic estimates (e.g. regression coefficient) AND variation (e.g. standard deviation) or associated estimates of uncertainty (e.g. confidence intervals)
☒	☐	For null hypothesis testing, the test statistic (e.g. *F*, *t*, *r*) with confidence intervals, effect sizes, degrees of freedom and *P* value noted<br>*Give P values as exact values whenever suitable.*
☒	☐	For Bayesian analysis, information on the choice of priors and Markov chain Monte Carlo settings
☒	☐	For hierarchical and complex designs, identification of the appropriate level for tests and full reporting of outcomes
☒	☐	Estimates of effect sizes (e.g. Cohen's *d*, Pearson's *r*), indicating how they were calculated

Our web collection on statistics for biologists contains articles on many of the points above.

Software and code

Policy information about availability of computer code

Data collection	Custom R codes were used for genomic analyses and simulations. Codes are provided in the Supplementary Codes file.
Data analysis	Shape analysis was performed with tpsDIG2 (version 2.31) and the geomorph R package (version 3.1.2). Approximate Bayesian Computation was carried out using the abc R package (version 2.1), and cline modeling was done using the R package hzar (version 0.2-5). Alignments of genomic sequences were done with the software NovoAlign (version 3.03.00). Alignment settings are provided in the Supplementary Codes file. Custom analytical R codes are available in the Supplementary Codes file. The latter involved the R package Rsamtools (version 2.2.1).

For manuscripts utilizing custom algorithms or software that are central to the research but not yet described in published literature, software must be made available to editors/reviewers. We strongly encourage code deposition in a community repository (e.g. GitHub). See the Nature Research guidelines for submitting code & software for further information.

Data

Policy information about availability of data

All manuscripts must include a data availability statement. This statement should provide the following information, where applicable:

- Accession codes, unique identifiers, or web links for publicly available datasets
- A list of figures that have associated raw data
- A description of any restrictions on data availability

All raw Illumina sequences, demultiplexed by site (and sampling period for the site M1) are available from the European Nucleotide Archive (accession numbers ERS4388731-ERS4388743) under the project PRJEB37366 (https://www.ebi.ac.uk/ena/browser/view/PRJEB37366). Raw genome-wide nucleotide counts for all sites (and temporal replicates) are provided on Dryad (doi:10.5061/dryad.c59zw3r67). Source data are provided with this paper.

Field-specific reporting

Please select the one below that is the best fit for your research. If you are not sure, read the appropriate sections before making your selection.

☐ Life sciences ☐ Behavioural & social sciences ☒ Ecological, evolutionary & environmental sciences

For a reference copy of the document with all sections, see nature.com/documents/nr-reporting-summary-flat.pdf

Ecological, evolutionary & environmental sciences study design

All studies must disclose on these points even when the disclosure is negative.

Study description	We used poolSeq whole-genome sequence data from 11 sites across the lake-stream habitat transition of the Misty Lake watershed (N approx. 56 individual per site) to estimate allele frequencies and perform a clinal analysis to shed light on reproductive isolation in this parapatric system.
Research sample	We use samples from natural threespine stickleback (Gasterosteus aculeatus) from 11 sites across Misty Lake and its inlet stream (Vancouver Island Canada). The sex of the specimens was not controlled; individuals were sampled until a sample size of around n = 50 was attained. The reason is that sex determination is not always unambiguous without dissection, which would have required lethal sampling (our sampling strategy was non-lethal). However, extensive previous stickleback work in the Misty system has shown that such haphazard sampling generally leads to relatively balanced sex ratios. In addition, all our analyses exlcuded the sex chromosome, so that the influence of differences among sites in the sample sex ratio should be negligible. All sampled individuals were adults. One reason is that in a speciation study focusing on gene flow and hybridization, genetic structure during the adult (reproductive) stage is most meaningful. Furthermore, during the season in which sampling occurred, juveniles are very small, so that a single spine clip (the standard non-invasive tissue sample used in our study) would not have yielded sufficient amounts of DNA for genetic analysis.
Sampling strategy	We aimed for a sample size of at least 50 fish for each of the 11 sites (the distance between the most distant sites is less than 2km), which is expected to be adequate for estimating allele frequencies from pool Seq (see Ferreti et al. 2013 and Gautier et al. 2013). Given our median read length of 103x across sites, combined with our number of fish per site, we consider our estimations of allele frequencies highly precise.
Data collection	Field sampling of all animals (for both morphometrics and sequencing) was conducted by KO with unbaited minnow traps. Sequencing libraries (DNA pools) were prepared by QH, and sequence data were generated by sequencing to high read depth on an Illumina HiSeq2500 instrument at the Quantitative Genomics Facility, D-BSSE, ETH Zürich in Basel.
Timing and spatial scale	Main field sampling at all 11 sites in the Misty Lake watershed occurred from the end of May to the beginning of July 2016. This is the classical season used to sample adult stickleback, thus making our work most comparable with the large body of research already available for this study system. For the marsh site M1, a second sample was taken during the flood on June 25 2016, and a third sample approximately one year later on August 3 2017. The total swimming (along-shore) distance between the most distant sample sites is approximately 1.8 km, and the distance between neighboring sample sites varies from around 45 to 600 m. Because the lake population was assumed to be genetically well mixed (confirmed by our data), we aimed for much more spatially dense sampling along the stream. At a given sample site, multiple traps were spread over a distance of around 20 m, and individuals were pooled across these traps. This conforms to the sampling procedure applied in previous research on Misty stickleback.
Data exclusions	Sequence data from the sex chromosome (Chr XIX) were excluded from analysis, as otherwise the results may have been influenced by slight differences among samples in the proportion of males and females.
Reproducibility	Because we took samples from natural populations subject to spatio-temporal changes in ecological conditions (e.g., the flood occurring during our sampling), the experiment cannot be reproduced in the strictest sense. However, apart from this stochasticity inherent in any natural system, our raw data available from the European Nucleotide Archive (project PRJEB37366), together with the Supplementary Software file, allow full reproducibility of our findings.
Randomization	The only grouping of individuals in our study is given naturally by the spatial sampling design. That is, individuals from a given sample site represent a group, and their DNA was pooled. Our study involves no treatments or other allocation of specimens to groups, hence randomization was neither necessary nor feasible.
Blinding	Field sampling and genomic analyses were performed by distinct teams of researchers. For the latter work, only sample IDs and a tissue sample were available, hence all genomic work was truly blind.

Did the study involve field work? ☒ Yes ☐ No

Field work, collection and transport

Field conditions	Field collections were performed under all weather conditions, because field work performed in the Misty system over many years by AH, KO, and DB has shown that stickleback catches are not influenced by weather conditions. The only exception is the flood; during a few days, sampling at stream sites was not feasilble for safety reasons.
Location	Misty Lake watershed, Vancouver Island, Canada (Latitude and Longitude are given in decimal degrees)

Site Habitat Latitude Longitude
L1 Lake 50.60507824 -127.2685989
L2 Lake 50.604347 -127.262569
M1 Swamp 50.60516595 -127.2579478
M2 Swamp 50.605087 -127.257812
S1 Stream 50.604618 -127.257198
S2 Stream 50.604414 -127.256683
S3 Stream 50.604375 -127.256141
S4 Stream 50.603808 -127.255397
S5 Stream 50.603056 -127.252444
S6 Stream 50.60223555 -127.2507798
S7 Stream 50.60060871 -127.2476535

This information is provided in the Supplementary Information (Supplementary Table 1)

Access and import/export	Field work was performed with permission from the following authorities to Andrew Hendry, McGill University:<br>- Fisheries and Ocean Canada, Species at Risk - License N° XRSF 14 2015, File N° SARA 368, issued 25 May 2015<br>- British Columbia, Ministry of Environment - Ecological Reserve Permit N° 102693, issued 30 October 2014<br>- British Columbia, Ministry of Forest, Lands and Natural Resource Operations - Fish Collection Permit N° NA16-225865, File 34770-20, issued 29 April 2016
Disturbance	The study did not cause any disturbance of the habitat.

Reporting for specific materials, systems and methods

We require information from authors about some types of materials, experimental systems and methods used in many studies. Here, indicate whether each material, system or method listed is relevant to your study. If you are not sure if a list item applies to your research, read the appropriate section before selecting a response.

Materials & experimental systems

n/a	Involved in the study
☒	☐ Antibodies
☒	☐ Eukaryotic cell lines
☒	☐ Palaeontology
☐	☒ Animals and other organisms
☒	☐ Human research participants
☒	☐ Clinical data

Methods

n/a	Involved in the study
☒	☐ ChIP-seq
☒	☐ Flow cytometry
☒	☐ MRI-based neuroimaging

Animals and other organisms

Policy information about studies involving animals; ARRIVE guidelines recommended for reporting animal research

Laboratory animals	The study did not involve laboratory animals
Wild animals	Adult wild stickleback fish (Gasterosteus aculeatus) of both sexes were caught with unbaited minnow traps. A dorsal fin was cut from each fish and stored immediately in ethanol. Fish were then released back into the field a the original collection site. At some location, a picture of the specimens was additionally taken for morphometric analysis. All sampling was thus non-lethal.
Field-collected samples	The study is based on tissue samples from wild animals only (see previous point); no live animals were taken to the laboratory.
Ethics oversight	All animal work in this study was conducted in accordance with the Animal Use Protocol from McGill University.

Note that full information on the approval of the study protocol must also be provided in the manuscript.
